# The New York Academy of Medicine

**Published:** 1880-01

**Authors:** 


### Reports of Societies.

### THE NEW YORK ACADEMY OF MEDICINE.

The Academy was called to order at 7:45 P.M., and the minutes of the last stated meeting were read by the Secretary, Dr. H. T. Hanks.

The annual report of the Treasurer was read, and showed a balance in the treasury of $342.03.

The report of the Board of Trustees was read by Dr. S. S. Purple, and showed a balance of $293.97 in favor of the Academy, and a total valuation of all the property belonging to the Academy of $81,400.

The annual report of the Recording Secretary was read, and showed an increase of 33 in the resident fellowship during the past year.

The report of the Corresponding Secretary was read by

Dr. John G. Adams, and a resumption of the publication of the volume of Transactions was recommended.

The Committee on Library reported, through the Librarian, Dr. Laurence Johnson, recommending a circulating department of the library and an additional reading room. The recommendations had been adopted by the council. The number of medical journals to be found in the library the coming year is 122. Of that number there are 24 German, 24 French, 12 English, 4 Spanish, 4 Italian, 1 Cuban, and 53 American.

The reading of the annual reports of other committees was dispensed with, and the Academy proceeded to the nomination of officers, after which a paper on

*Some of the Commoner Affections of the Tonsils, from a Diagnostic and Therapeutic Standpoint,* was read by Dr. Geo. M. Lefferts. (Vid. page 601.)

The paper being before the Academy for discussion,

Dr. J. H. Douglas referred to one point not mentioned by Dr. Lefferts, and which he thought of considerable importance in studying affections of the tonsils—namely, the situation of the tonsil between the arches of the palate, anterior and posterior, which in the acute stage of inflammation strangulated the gland to a greater or less degree, thus tending to keep it filled with blood, perpetuate the pain, and prolong the disease.

In chronic enlargement of the tonsils the anatomical arrangement and the effect were the same, and in most cases the strangulation was very marked, which was kept up by the catarrhal inflammation.

The practical point was to relieve the catarrhal inflammation in the acute cases, especially affecting the posterior half of the posterior arch and nasal fossa, and that could be done best by injecting those parts forcibly by means of a syringe with a long, curved nozzle, so as to remove the masses of thick mucus which had lodged there. When that was done from four to six times daily, the tumefaction of the tonsil usually at once subsided, the patient was relieved, and was able to fall asleep readily.

In chronic tonsilitis he resorted to the same plan of treatment, together with washing out the crypts of the tonsils with water and keeping them free from accumulations of secretion. When that was done, it was remarkable with what celerity the hypertrophy would disappear, and the treatment followed up from time to time had reduced the largest tonsil he had seen. He did not approve of the use of nitrate of silver, and he now rarely used a knife. With regard to hemorrhage following excision of the tonsils, he thought there would be no difficulty if the coagulum was kept thoroughly removed. He agreed with Dr. Lefferts upon the point that quinsy sore-throat occurred for the most part in those who had an arthritic constitution.

Dr. Beverley Robinson directed attention to one practical point in the differentiation of follicular tonsilitis from diphtheria. Both were contagious; but he believed that when the membrane fell off in follicular tonsilitis, it did not reappear, as was the case in diphtheria, two or three days after falling of the membrane. Albumen also was present in the urine of the diphtheritic patient, while in cases of follicular tonsilitis it was absent.

He agreed with Dr. Lefferts regarding the inefficiency of gargles, the benefits from inhalation, the uses of scarifications, and incisions in the acute affections of the tonsils. He also corroborated what had been said regarding the evils attending chronic inflammation of the tonsils, and partially agreed with him regarding excision.

He was disposed to believe that follicular or herpetic tonsillar disease had simply a different localization, but was the same disease as inflammatory or membranous croup.

Dr. D. B. St. John Roosa remarked that the speaker was not willing to state positively whether the tonsil actually pressed upon the Eustachian tube, thus producing deafness, or not. He believed, however, that an inflamed tonsil never pressed upon the mouth of the Eustachian tube so as to cause deafness; but that the deafness, if present, was coincident, and depended upon inflammation of the mouth or calibre of the tube or tympanic cavity, caused by the same conditions as those which gave rise to the tonsillar affection. He was sorry for the introduction of the term "throat deafness," because it implied that there was a form of deafness dependent upon disease of the throat. In his opinion there was no deafness depending upon disease of the throat, unless the Eustachian tube or the tympanic cavity, as well as the throat, was involved. In acute tonsilitis he thought impairment of hearing very seldom was present, unless there was the history of a catarrhal deafness preceding the acute attack. He believed that we might have never so much swelling of the tonsils, and yet no impairment of hearing. Nevertheless, enlargement of the tonsils became an important factor in the treatment of diseases of the ear, and the ear could not be put into a healthy condition so long as such enlargement existed. Therefore, otologists were accustomed to advise excision of the enlarged tonsils, in order to favor obtaining a healthy throat, which so much favored a healthy ear.

With reference to gargling, he was in full accord with Dr. Lefferts regarding its inefficacy; yet he believed it might be made much more efficacious than it ordinarily was, by following von Troltsch's method, which had not received the consideration to which it was entitled.

VON TROLTSCH'S METHOD OF GARGLING.

The method is as follows: The gargle is held in the back part of the mouth, the head thrown well back, and the nostrils closed with the fingers; swallowing motions are then performed without actually swallowing the solution. That by this method the pharynx could be reached he had proven by painting the wall of the pharynx with iodine and then causing a solution of starch to be used as a gargle, when the characteristic reaction was produced invariably.

Dr. R. P. Lincoln remarked with reference to the wide influence which diseased tonsils, especially chronic hypertrophy, exerted over the ears, that it had been his fortune, in several instances, to see quite anomalous phenomena, which had led him to seek for a cause for ear-phenomena associated with tonsillar affections, and he had reached the conclusion that it was purely mechanical, and produced through the agency of the nerves coming from the glosso-pharyngeal, acting in a reflex manner. There was also, through the otic ganglia of the sympathetic system, a connection between the throat and the ear. Within the past week, in endeavoring to remove a collection of inspissated mucus from a tonsil, although it was done with the very least degree of violence, the patient subsequently suffered from pain in the ear sufficiently severe to prevent sleep, and yet there was no manifestation whatever of inflammatory action in the ear itself. He thought such collections of inspissated mucus in the tonsils required treatment much oftener than, perhaps, ought to be inferred from the remarks of the writer of the paper. There were many patients who had enlarged tonsils, not perhaps to a degree to require excision, but often requiring treatment because of the pharyngeal catarrh they produced, and such cases he believed should be treated by applications, such as referred to, into the crypts themselves of the tonsils. Perhaps nitrate of silver might be used. A favorite mixture with him was carbolic acid and co. tr. of iodine, in the proportion of 1 to 2, applied directly to the lining membrane after the crypts had been rid of the collections of mucus. It was not always successful; and, when not, excising a small portion and applying the galvano-cautery to the lining of the crypt would almost always effect a cure.

He referred to a case in which a collection of inspissated mucus was as large as an English walnut.

Dr. Bosworth believed that the exudation in follicular tonsilitis was croupous in character, and that it could be best cured by passing a probe coated with nitrate of silver into the crypts and the administration of large doses of quinine. With regard to the many evils attending enlarged tonsils, such as poisoned air going to the lungs, dyspepsia, etc., he was in accord with Dr. Lefferts, but with reference to the sinking in of the bones of the chest and the formation of "pigeon-breast," referred to by Dr. L. as one of the results of enlarged tonsils, he should simply attribute that condition to a rachitic diathesis preceding and probably favoring the existence of enlarged tonsils. He also referred to nightmare as a common sympton of chronic enlargement of the tonsils.

With regard to *acute tonsilitis*, he thought it quite probable that, in most cases, no form of abortive treatment possessed special efficacy; still, in a number of instances he had succeeded in aborting what he believed was the disease by giving grs. x. of quinine, and following it with Fleming's tincture of aconite in such doses as would rapidly produce marked physiological effects of the drug, such as dryness of the throat, dizziness, and ringing in the ears, and then discontinue it. That method would do no good if suppuration had been established.

He regarded croupous, catarrhal, and diphtheritic as three distinct forms of inflammation, and thought that more care should be taken in making the nomenclature of diseases of throat to correspond to the pathological state.

Dr. A. H. Smith thought that incising the glands in *acute tonsilitis* left wounds nearly as large as would be made by ablation, and as the latter was the most efficient, it might better be employed at once.

With regard to occasional *failure of the stump of the tonsil to atrophy* after the projecting portion had been removed, it was perhaps to be explained, at least in some cases, that the tonsil receives blood at its upper portion, and the lower portion alone has been removed. If the upper portion of the tonsil was cut off, the supply of blood was removed, and the operation was quite effectual. If the anterior pillar of the fauces was adherent to the tonsil, as it was not infrequently, it was important to first break up the adhesions, so as to secure complete ablation of the superior portion of the gland.

He suggested the following method of *coating a probe with nitrate of silver:* Dip the extremity of the probe into powdered nitrate of silver and then hold it for an instant over a flame, and the silver that adheres to the probe will melt and form a smooth covering.

Dr. F. A. Burrall remarked, regarding the treatment of tonsilitis, that he did not accept the views of those who dissented from the use of *gargles*, because they did not reach the pharynx; for, as a rule, in tonsilitis it was not the pharynx we wished to reach. A gargle might be used so as to give pain, or it might be a temporary substitute for a poultice and give efficient relief. The action of a warm gargle repeated every half-hour was to favor suppuration, and, when they contained emollient substances, were very soothing and beneficial. Gargles, steam, and spray, all had their value. Stimulating gargles, were of value in reducing the size of hypertrophied tonsils. He favored constitutional treatment in throat diseases, and by preference gave a calomel purge in the early stages of acute tonsilitis.

All acute sore throats should be treated as if infectious, especially in children.

Tonics, beginning very early, and frequency of application, were very important in the abortive treatment of tonsilitis and other forms of sore throat.

REPORT OF THE STATISTICAL SECRETARY.

Dr. F. V. White, Statistical Secretary, announced the death of Freeman J. Bumstead, M.D., L.L.D., and gave a brief sketch of his life.

The President paid the following tribute to his memory:

By the death of Dr. Bumstead, the Academy of Medicine and the profession of this city have lost one of its most eminent men, who contributed largely to give it the rank which is now conceded to it, not only in this country but abroad. He had always been a most industrious, methodical, able and honest worker for its science and its literature, and the results of his labors will hold a prominent and a permanent place in medical literature.

As an intimate acquaintance of more than twenty-five years, I am competent to say of his personal character, that it secured the warm affection and sincere esteem of all those who enjoyed this privilege.

In accordance with the by-laws, I will designate to prepare a memoir of Dr. Bumstead, one eminently qualified to do justice to his personal qualities, his professional abilities, and his literary work—Dr. Geo. A. Peters.

ADOPTION OF NEW BY-LAW.

The new by-law relating to fellowship was adopted to take effect after the first meeting in January, 1880. The Anniversary Meeting was announced to take place on Thursday evening, December 11th, with Dr. Leroy M. Yale as orator, and his subject, "The Academy as an Educator."

The Academy then adjourned.

---

ANNIVERSARY MEETING, DECEMBER 11, 1879.

Fordyce Barker, M.D., L.L.D., President, in the Chair.

The Academy was called to order at 8 P.M., and prayer was offered by Rev. Dr. Washburn.

The orator of the evening, Dr. Leroy M. Yale, being unavoidably detained at home on account of sickness, Dr. F. A. Castile read the oration, which had for its subject

"THE ACADEMY AS A TEACHER."

A brief reference was made to the early history of the Academy in comparison with its present position in its new library hall, the munificent representation of the far-sightedness of one of the fellows; to the methods of controlling the education preparatory to entering the medical profession; to the great law of supply and demand, which had a marked influence in every department of society to the principle that men, like water, found their true level—a principle which pretty constantly affected every member of society; and then the orator passed to the main subject of the oration, and inquired how had the Academy taught in the past, how is she teaching, and what part will she take in the teaching of the future?

The Academy had been efficient as a teacher in two important ways—namely, cultivation of the science of medicines, and elevation of the character and honor of the profession.

With reference to the first, the scientific papers presented during the thirty years it had existed, possessed considerable value; indeed, as has been said, "a value far greater than we can well imagine." It might be said that those old papers had been left far behind, and were worthless as aids to modern study; but their value must be estimated from the standpoint and the date at which they were writen, and then their intrinsic worth became apparent, and the influence which they exerted upon the education of those who lived subsequent to their authors might be duly appreciated.

The second avowed object in founding the Academy was an ethical one, and that end had not been lost sight of. The loss of the esteem of the fellows was a potent factor in regulating the ethics of the profession, in elevating the character of its members, and maintaining its honor; and it was to be hoped that the influence of the traditions of the fathers would not be lost in this particular—namely, only those were elected to fellowship who won the position by purity of conduct.

The orator then referred to the library of the Academy, its origin and its work, and urged its claims upon the fellows and the profession for contributions of books, even old books—for it was nothing of disparagement to a library that it was rich in old books; and the calm wisdom of old experience was oftentimes more substantial aid than the brilliant exploits of young enthusiasm. The Academy had opened its reading-rooms, and the influence which a storehouse of books could exert might now permeate among the reading classes. But there were others whose wants should be supplied, and to that end he urged, with Dr. Thomson, who delivered the Anniversary Address for 1878, that lectureships should be established; and, in order to secure the lectures, if funds were not forthcoming, an appeal should be made to those who are able to lay aside their modesty and speak without pecuniary reward.

He also suggested the founding of scholarships, and asked if it was not possible for the Academy to found a museum. If museums were good, there could not be too many of them. To be sure, the city had Wood's Museum at Bellevue Hospital—a monument of great personal energy and industry; the New York Hospital Museum, and college museums. But the museums of hospitals and colleges would always be increased by those who were in close affiliation with them, while a museum founded by the Academy of Medicine would gather a new type of specimens, and a large amount of valuable material which now was wasted. A museum, once started, could be built up the same as has been the library, and all that could illustrate the science and art of medicine could be secured for the Academy, if it would undertake to be its custodian.

In conclusion, the orator alluded to the Academy as an association not devoted to special study or narrow special ends, but a common meeting-ground of the best elements of our professional body, and an organization that had the power of age and the character of authority.

The oration was well received.

The President extended the thanks of the Academy to Dr. Castle, after which the exercises were closed by the benediction.—*Medical Record.*

